# Is There an Effect of Fetal Mesenchymal Stem Cells in the Mother–Fetus Dyad in COVID-19 Pregnancies and Vertical Transmission?

**DOI:** 10.3389/fphys.2020.624625

**Published:** 2021-02-11

**Authors:** Athina Samara, Eric Herlenius

**Affiliations:** ^1^Department of Women’s and Children’s Health, Karolinska Institutet, Stockholm, Sweden; ^2^Astrid Lindgren Children′s Hospital Karolinska University Hospital, Stockholm, Sweden

**Keywords:** COVID-19, SARS-CoV-2, neonate, pregnancy, vertical, mesenchymal, MSCs

## Abstract

Because of the polysystemic nature of coronavirus disease 2019 (COVID-19), during the present pandemic, there have been serious concerns regarding pregnancy, vertical transmission, and intrapartum risk. The majority of pregnant patients with COVID-19 infection present with mild or asymptomatic course of the disease. Some cases were hospitalized, and few needed intensive care unit admission, or mechanical ventilation. There have also been scarce case reports where neonates required mechanical ventilation post COVID-19 pregnancies. Without approved therapies other than dexamethasone, advanced mesenchymal cell therapy is one immunomodulatory therapeutic approach that is currently explored and might hold great promise. We suggest that the circulating fetal stem cells might have an immune-protective effect to mothers and contribute to the often mild and even asymptomatic post-COVID-19 pregnancies. Thus, COVID-19 pregnancies come forth as a paradigm to be further and more comprehensively approached, to understand both the mechanism and action of circulating stem cells in immunoprotection and hypoxia in microcirculation.

## Introduction

Maternal–fetal transmission of viral diseases may occur transvaginally or through the hematogenic, i.e., the transplacental transmission pathway. In the latter, the virus circulating in the maternal blood vessels may reach and enter the placenta across chorionic villous and non-villous structures fetal blood vessels and be transmitted to the fetus. This mechanism of vertical transmission was not reported after the infection of pregnant women with the coronaviruses, severe acute respiratory syndrome coronavirus 1 (SARS-CoV-1) and Middle East respiratory syndrome coronavirus (MERS-CoV). Despite the fact that pregnant women may be infected by these coronaviruses other than severe pneumonia, they may face complications such as early pregnancy loss or even death (Schwartz and Graham, 2020).

The uterine enlargement during pregnancy is known to bring major changes in maternal physiology. These can be both mechanical, such as reduced functional residual volumes and diaphragm elevation, and cellular, such as altered cellular immunity. The maternal immune system may tolerate fetal antigens suppressing cell-mediated immunity while retaining normal humoral immunity, changes known to occur locally at the maternal–fetal interface, which could affect systemic immune responses to infection (Jamieson et al., 2006). This may render pregnant women more vulnerable to viral infections, especially in cases where the infections may have an effect on the cardiorespiratory system during pregnancy and could enhance progression to respiratory failure (Dashraath et al., 2020). The ability to increase ventilation is reduced, when pregnant. Therefore, there is an increased risk of inadequate response to environmental stressors such as upper/lower respiratory tract infections and hypoxic and hypercapnic respiratory failure (Lapinsky et al., 2014). During the SARS epidemic, up to 35% of the infected pregnant patients required mechanical ventilation, and mortality rates reached 18%; in the case of MERS, the numbers reached 41 and 25%, respectively (Dashraath et al., 2020; Liu et al., 2020; Schwartz and Graham, 2020).

## SARS–Coronavirus 2 in Pregnancy

Coronavirus disease 2019 (COVID-19) is a polysystemic disease, aggravated by compromised immune response, increased body mass index, and other comorbidities. Thus, there have been serious concerns regarding the pregnancy and the vertical transmission and intrapartum risk (Ferrazzi et al., 2020; Gupta et al., 2020).

The majority of pregnant patients with COVID-19 infection present with a mild or asymptomatic course of the disease (Ferrazzi et al., 2020). Some cases were hospitalized, and few needed intensive care unit admission or mechanical ventilation; some patients received oxygen support, whereas others were treated with antibiotics, antivirals, systemic corticosteroids, and other treatment combinations (Breslin et al., 2020; Chen H. et al., 2020; Dashraath et al., 2020; Friedman et al., 2020; Hecht et al., 2020; Iqbal et al., 2020; Liu et al., 2020; Oncel et al., 2020; Savasi et al., 2020; Takemoto et al., 2020), while several clinical trials are ongoing to evaluate the choice of treatment in pregnant or breastfeeding COVID-19 patients (Dashraath et al., 2020; Pastick et al., 2020). There have also been some case reports where neonates required mechanical ventilation post-COVID-19 pregnancies (Alwardi et al., 2020; Amaral et al., 2020; Gale et al., 2020; Gregorio-Hernández et al., 2020; Kirtsman et al., 2020; Oncel et al., 2020; Savasi et al., 2020). The studies that concern maternal–fetal transmission of virus have some limitations. First, analyses of SARS coronavirus 2 (SARS-CoV-2) in newborns are often delayed; thus, there is an increased risk of extrauterine transmission. Second, the majority of the present case reports include the analysis of late vaginal smears (Zeng L. et al., 2020), instead of the sampling of amniotic fluid. The sampling of amniotic fluid could have provided an indication that vertical transmission might have occurred. A few studies investigated SARS-CoV-2-positive placental sampling to document direct viral involvement or vertical transmission (Alamar et al., 2020; Schwartz and Morotti, 2020; Smithgall et al., 2020; Taglauer et al., 2020). Although there have been case reports of histomorphologic evidence of maternal/fetal vascular malperfusions (Smithgall et al., 2020), there is still no concrete proof that SARS-CoV-2 placental invasion may lead to fetal pathology.

Different studies have shown that prevalence of COVID-19 test positivity among small studies of neonates vary, from less than 1% and up to 5% (Ashraf et al., 2020; Dumitriu et al., 2020; Khoury et al., 2020; Remaeus et al., 2020; Verma et al., 2020; Walker et al., 2020). Meanwhile, several study groups are actively investigating the subject of SARS-CoV-2 vertical transmission of infection, focusing on sample collection as early as possible and ideally within the first 12 h after birth. One of them is the periCOVID (COVID-19 Clinical Research Coalition, 2021) study, set up in the United Kingdom under the umbrella of Public Health England, also recruiting in Africa. The primary objective of the periCOVID surveillance study is to assess the risk of COVID-19 vertical transmission and identify and determine the routes, collecting samples from breast milk, placenta, and cord blood at birth for reverse transcriptase–polymerase chain reaction (RT-PCR) and sequencing followed by sequential sampling of maternal and neonatal urine and feces and blood samples for serology.

Some case reports and small studies have indicated that maternal–fetal transmission might occur (Table 1). They report different combinations of positive RT-PCR testing of neonatal nasopharyngeal swabs at birth followed with negative serology and later seroconversion of the mother (Alzamora et al., 2020), negative RT-PCR results of nasopharyngeal swab (Dong et al., 2020; He et al., 2020; Zeng H. et al., 2020) and positive neonatal blood serology immediately after birth, and several other combinations of COVID-19 diagnostic results. Unfortunately, neither amniotic fluid and placenta nor cord blood was tested in most cases. Immunoglobulin M (IgM) against SARS-CoV-2 proteins has been detected in some newborn case reports. Thus, as IgM does not cross the placental barrier, it raises the possibility of vertical transmission of the virus leading to IgM production by the fetus. However, this is not conclusive evidence and may also be due to placental alterations allowing the passage of IgM, or false-positive testing.

**TABLE 1:** List of publications providing information on laboratory tests of mothers and neonates who tested positive with COVID-19.

Author and publication date	Pregnant women (*N*)	Neonates (*N*)	Mother								Neonate												Pregnancy			
			Naso pharyngeal swab		Serum		Breast milk		Vaginal swab		Nasopharyngeal swab or Deep Trachea		Blood Sample		Gastric Juice		Anal swab/Stool		Urine Sample		Umbilical Cord blood		Amniotic fluid		Placental Tissue	
			+	−	+	−	+	−	+	−	+	−	+	−	+	−	+	−	+	−	+	−	+	−	+	−
Wang et al., 2020	1	1	1					1			1											1				1
Dong et al., 2020	1	1	1		1			1		1		1	1													
Zeng L. et al., 2020	6	6			5							6	5													
Nie et al., 2020	27	28	27								1	25										1				1
Carosso et al., 2020	1	1	1		1					1		1	1				1				1					1
Zamaniyan et al., 2020	1	1	1							1	1											1	1			
Baud et al., 2020	1	1	1									1		1											1	
Buonsenso et al., 2020	2	2	2									2	1	1							1	1			1	1
Hosier et al., 2020	1	1	1		1																1				1	
Govind et al., 2020	9	9	9								1	8														
Piersigilli et al., 2020	1	1	1								1															
Penfield et al., 2020	32	32	32									11													3	8
Patanè et al., 2020	2	2	2								2														2	
Costa et al., 2020	1	1	1				1														2				2	
Schoenmakers et al., 2020	1	1	1					1				1													1	
Kirtsman et al., 2020	1	1	1				1		1		1		1												1	
McDevitt et al., 2020	8	8	1	7							8															
Ferraiolo et al., 2020	1	1	1		1							1													1	
Hu et al., 2020	7	7	7								1	6		7				7		7						
Gao J. et al., 2020	64	64	24									24	11								1					
Nayak et al., 2020	141	131	141								3	128														
Pulinx et al., 2020	1	2	1		1																		2		2	
Vivanti et al., 2020	1	1	1		1				1		1		1				1						1		1	
Rivera-Hernandez et al., 2020	1	1	1								1															
Peng et al., 2020	1	1		1	1							1	1													
Richtmann et al., 2020	5	5	5																					2	2	3
Farsi et al., 2020	1	3	1								1	2													1	
Sisman et al., 2020	1	1	1								1														1	
Khoury et al., 2020	241	236	241								6	230														
Hecht et al., 2020	19	19	19								1														2	
Savasi et al., 2020	77	57									4															
Lorenz et al., 2020	1	1	1								1						1									
Sun et al., 2020	3	3	3								3															
Algarroba et al., 2020	1	1										1													1	
Kulkarni et al., 2020	1	1		1	1						1										1				1	
Oncel et al., 2020	125	125						6			4	116		3				2						4		5
Toner et al., 2020	1	1	1		1							1		1							1			1		
Taglauer et al., 2020	15	15									12														15	
Hinojosa-Velasco et al., 2020	1	1	1				1				1															
Vendola et al., 2020	2	2	1	1	2							2	2								2		1			
Mongula et al., 2020	1	1	1					1				1													1	
Vashukova et al., 2020	6	6	6									6													1	5
Facchetti et al., 2020	1	1									1														1	
Anand et al., 2021	69	65									7															
Gao W. et al., 2020	1	1	1		1							1	1												1	
Menter et al., 2020	5	5	5									5													2	3
Sagheb et al., 2020	2	2	1								2															
Zhou et al., 2020	16	16	1								1		1													
Cavaliere et al., 2020	1	1	1								1		1													
Fenizia et al., 2020	30	30	30		19		1		1		2										12				2	28
Bachani et al., 2020	348		57								5															
Mattar et al., 2020	16	5	16		5																5					5
Adhikari et al., 2020	252	188	252								6	182														
Hsu et al., 2021	1	1	1									1													1	
Alamar et al., 2020	1	1	1								1														1	
Stonoga et al., 2020	1	1	1								1										1		1		1	
Rodrigues et al., 2021	1	1	1								1															
Parsa et al., 2020	1	1	1								1												1			
Di Mascio et al., 2020	251		251								1															
Sileo et al., 2020	1	1	1									1									1					
Pessoa et al., 2020	1	1									1															
Alwardi et al., 2020	1	3	1								3															
Hcini et al., 2020	507	127	137		46/76						4	108														
He et al., 2020	22	22											11													
Debelenko et al., 2020	75	75	75									75									1				1	
Zaigham et al., 2020	1	1	1		1						1		1												1	
Shende et al., 2020	1	1	1																				1		1	
Bandyopadhyay et al., 2020	1	1	1								1		1													
Edlow et al., 2020	127	1	64								0		1	76							1					88

As expected, SARS-CoV-2 RT-PCR-positive diagnosed mothers with IgG antibodies against SARS-CoV-2 transfer these to the fetus (Gao J. et al., 2020). However, there have also been some reports where IgM were detected in the fetus. These include case reports and small studies from China [2 preterm (Dong et al., 2020; Wu et al., 2020d), 1 term (Zhou et al., 2020), 2 term, 10 term (Gao J. et al., 2020), 2 preterm-1 term (He et al., 2020)], the United States [one term (Edlow et al., 2020)], Italy [one preterm (Fenizia et al., 2020), one term (Cavaliere et al., 2020)], and Sweden (three term Herlenius et al. at Karolinska University Hospital, Stockholm, in progress) that also exhibited IgG against SARS-CoV-2 nucleocapsid and S-protein antibodies 0–2 days after birth.

## SARS-CoV-2 Compromised Immunity

Because of the unprecedented participation of volunteers, supported by huge private and governmental investments, both phase III clinical trials of the vaccines developed by BioNTech/Pfizer and Moderna concluded in November 2020. At the time of writing, Pfizer’s vaccine was approved for emergency use in the European Union, United Kingdom, Canada, and the United States, whereas Moderna’s vaccine was also approved for use in the United States. However, pregnant women were excluded from the vaccine clinical trials; thus, the vaccines were not authorized for use during pregnancy.

Vaccination plans take into account several factors, among which are age and comorbidities. Not only progression in COVID-19 patients but also percentages of asymptomatic prevalence vary considerably. A proof-of-principle example of this comes from a study from Stockholm, Sweden, where among patients presenting in labor at Karolinska University Hospital from March 25 to July 24, 2020, 65% of those diagnosed as RT-PCR-positive were asymptomatic (Ahlberg et al., 2020); a similar small study between February and March 2020 in Stockholm showed that 31% of the children reporting no symptoms were seropositive (Herlenius et al., 2020).

However, the highest COVID-19-related morbidity is observed in the group of old-age patients with co-morbidities. Because of their age and medical history, both the immune and the tissue regenerative capacity of these patients are compromised, resulting in this subgroup being affected the most by coronavirus.

Old-aged and other critically ill patients suffer the effects of the aberrant systemic inflammatory response known as the cytokine release syndrome, or the infamous “COVID-19 cytokine storm.” This cytokine storm clinically presents with a sharp rise of cytokines within a short time and may, among others, cause lung injury, which in turn may progress into acute lung injury or its more severe form, acute respiratory distress syndrome (ARDS). The exact pathology and the mechanism of lung injury proceeding to respiratory failure and ARDS in COVID-19 patients are not yet fully understood; yet, it leads to low oxygen saturation levels. Overproduction of proinflammatory cytokines is one of the parameters and a major cause of mortality in COVID-19 (Chen N. et al., 2020; Huang et al., 2020; Lai et al., 2020).

Coronavirus disease 2019 disease progression presents with distinct symptoms, suggesting diversified host immune responses, with one of the villains being the dysfunctional interferon system. A recent study showed that excessive inflammatory response in severe and critical patients was associated with persistent viremia associated with the expression of the nuclear transcription factor nuclear factor-κB; patients presented with type I interferon deficiency, increased production, and altered signaling of tumor necrosis factor-α and interleukin 6 (IL-6; Hadjadj et al., 2020). Such data suggest that combined therapeutic approaches, which could alleviate severe COVID-19 and hasten recovery of the patients who are critically ill, are vital.

One of the main challenges when treating COVID-19 is that the documented exacerbated anti-SARS-CoV-2 immune response can cause severe disease if it remains uncontrolled. The cytokine storm is this inflammatory cascade associated with this exaggerated response of innate immunity, which might cause a late or ineffective response of adaptive immunity (Ragab et al., 2020). It seems to lead to dysfunction of the microcirculation that becomes the villain of SARS-CoV-2 infection (Colantuoni et al., 2020). Thus, a number of therapies aim to target the immune system, but dexamethasone is so far the only drug that can ameliorate disease outcome also in intubated patients (RECOVERY Collaborative Group et al., 2020). Dexamethasone has a long half-life, acts on glucocorticoid receptors, and reduces inflammation through a broad-pathway approach that has been associated, among others, with immunosuppression, hospital-acquired infections, and neuromuscular weakness, even with short courses. Monoclonal antibody therapies of Eli Lilly and Regeneron have also been granted emergency use approval in the United States, for use in mild and moderate cases. IL-6 antagonists have not yet been proven successful (Scherger et al., 2020).

The attenuated deficient immune system is the major risk factor in COVID-19. However, pregnant women also have an attenuated immune system, due to the fetal allograft, but they do not display increased vulnerability under COVID-19. But it might be that there is, currently explored, one immunomodulatory therapeutic approach that might hold great promise toward answering this question. That is advanced mesenchymal cell therapies, i.e., the application of stem cells to treat patients with COVID-19 (Saldanha-Araujo et al., 2020).

## Mesenchymal Stem Cells

Exogenous mesenchymal stem cells (MSCs) have been used for decades to treat other diseases caused by viruses. Examples include the acute lung injury caused by influenza virus the immunological compromises caused by the human immunodeficiency virus and the chronic hepatitis caused by hepatitis B virus (Thanunchai et al., 2015). MSCs are a type of highly proliferative adult stem cells with multilineage differentiation capacity. Initially isolated from the bone marrow, they subsequently identified in other tissues, such as the dental pulp, umbilical cord and placenta, adipose tissue and even periosteum and skeletal muscle. They can be found in various autologous and allogenic sources.

Several lines of evidence indicate that one of the main therapeutic mechanisms of MSC administration is via immunomodulation. MSC-mediated immunomodulation operates through a cohort of cell contact-dependent mechanisms including gap junctions and soluble factors (Wu et al., 2017; de Witte et al., 2018; Naji et al., 2019). The main advantage of the use of MSCs for clinical research lies with their hypoimmunogenicity, and they are thus known as “immune-privileged cells.” They do not express human leukocyte antigen (HLA) class II molecules or costimulatory molecules such as CD40, CD40L, CD80, and CD86 and express low levels of HLA class I molecules. These characteristics permits the MSCs to escape the cytotoxic effects of lymphocytic T cells, B cells, and natural killer cells (Rasmusson, 2006; Stagg, 2007; Weiss, 2014; Can and Coskun, 2020; Li et al., 2020). Furthermore, they are able to detect injury signals in their microenvironment and signal regeneration (Stappenbeck and Miyoshi, 2009; Le Blanc and Mougiakakos, 2012; Qin and Zhao, 2020).

Not only MSC-secreted cytokine-mediated effects but also apoptotic, metabolically inactivated, or even fragmented MSCs were shown to exert an immunomodulatory effect, but the roles of regulatory T cells and monocytes in the equation remain under investigation (see review Weiss and Dahlke, 2019). The MSCs’ immunomodulatory properties affect proliferation, activation, and function of various immune cells (Harrell et al., 2019) and may thus alter the innate and adoptive immune responses (Li et al., 2016). The underlying cellular and molecular mechanisms of the long-term effects of MSC-mediation are yet not fully clarified, but regenerative and immunomodulatory effects have been observed in various diseases and tissue types (Aguiar et al., 2020; Qin and Zhao, 2020; Sharma et al., 2020; Tao and Chen, 2020). Although studies describe MSCs as short-lived (Eggenhofer et al., 2012), unable to cross the lung capillary network after intravenous infusion (Fischer et al., 2009), there have been preclinical reports that survival of exogenous MSCs could be detected on site up to 4 months after their direct transplantation; however, few of these cells developed the tissue phenotype of the resident cells (Muñoz et al., 2018).

Mesenchymal stem cells may also use a connexin-43-dependent mechanism to aid compromised cells via mitochondrial transfer, increasing their survival (Islam et al., 2012). Older and recent studies have documented that MSC transplantation and transdifferentiation (Dilger et al., 2020) is dependent on gap junctional cell coupling and the intricate interplay and changes in expression levels of connexin family members. Pannexins might also be involved (Swayne et al., 2020) and thus MSCs might modulate the inflammasome via gap junctional dependent mechanisms.

Mesenchymal stem cells can also suppress chronic inflammation and promote tissue regeneration via the secretion of exosomes, which may in turn regulate macrophage polarization (Ti et al., 2015). Their extracellularly secreted vesicles (termed EVs, which includes both exosomes and microvesicles) are exploited as a cell-free therapeutic tool due to their paracrine and/or endocrine effects (Meirelles Lda et al., 2009). Their mode of action involves either binding to extracellular receptors of targeted cells, merging with the membrane and secreting EV contents, or entering the target cell as endocytic vesicles. MSC-derived EV therapies come with further clinical advantages as they pass across small blood capillaries due because of their small size; they have low chances of tumor formation as they are non-proliferative, and they are immune privileged as they are HLA-I and HLA-II negative.

## COVID-19 and MSCs

Because of these immunomodulatory properties of MSCs, 66 stem cell therapy clinical protocols against SARS-CoV-2/COVID-19 have been registered, and six trials have now been completed (details are available at www.clinicaltrials.gov). The use of stem cells as possible therapeutic approaches against COVID-19, including not only MSCs but also MSC-derived exosomes, as therapeutic opportunities for COVID-19 has gained momentum (Jacob et al., 2020; Jayaramayya et al., 2020). As mentioned, most intravenously injected MSCs seem to get trapped in the lung. However, their role in the injured lung may be anti-inflammatory and antiviral and aiding in tissue repair utilizing cell-to-cell contacts and without engraftment into the damaged tissue (Galleu et al., 2017; Armitage et al., 2018), But in addition, and more importantly, *in vivo* MSCs survive in a hypoxic niche where oxygen tensions are oftenless than 10%.

Many of the protocols submitted to treat COVID-19 involve MSCs derived from the umbilical cord and Wharton jelly. It is known that fetal MSCs are more potent in comparison to those derived from adult sources, but there are ethical considerations regarding fetal tissue as a cellular source. However, perinatal tissues are easily accessible for isolation, highly abundant, and not posing any major ethical concerns. More importantly, a number of clinical trials documented no adverse events after the infusion of MSCs; thus, these cells are considered safe for clinical use (Lalu et al., 2012). MSCs from perinatal tissues have stronger immunomodulatory properties than adult bone marrow-derived MSCs (BM-MSCs; Li et al., 2014). Therefore, MSCs isolated from umbilical cord (UC-MSC) and the fetal part of the placenta (PL-MSCs) share many similarities with BM-MSCs. Compared to adult BM-MSCs, UC-MSCs are superior in colony-forming capacity and differentiation potential; PL-MSCs have a lower colony-forming capacity but similar or less differentiation potential (Beeravolu et al., 2017).

## Discussion

Mesenchymal stem cell-induced modulation of immune response in COVID-19 patients seems to be a feasible therapeutic treatment. Currently (December 21, 2020), results from seven single case studies and five small studies (7, 9, 12, 16, and 27 patients, respectively) have been published, where MSCs were employed as therapy on COVID-19 patients (Feng et al., 2020; Liang et al., 2020; Meng et al., 2020; Scherger et al., 2020; Sengupta et al., 2020; Shu et al., 2020; Soler Rich et al., 2020; Tao et al., 2020; Yilmaz et al., 2020; Zengin et al., 2020; Zhang et al., 2020). The phase I clinical trial with the 27 patients (Wu et al., 2020c) with COVID-19 followed the first case study (Wu et al., 2020a) of infusion of MSC-like cells [called human embryonic stem cell-derived immunity- and matrix-regulatory cells (Wu et al., 2020b)] that show higher immunomodulatory potential than standard adult MSCs. These results, although from few patients, suggest that intravenous infusion of MSCs in COVID-19 patients does not cause severe side effects. In addition, MSC transplantation or MSC-derived exosome transplantation is associated with reduced inflammation, also in critically and severely ill COVID-19 patients (*n* = 70). Interestingly, a case study showed that MSC transplantation, also ameliorated and treated the central nervous system infection, and the authors propose simultaneous systemic and intrathecal administration, as MSCs can thus cross the blood–brain barrier (Yilmaz et al., 2020). Moreover, the outcome after stem cell administration included improvement in lung function. Therefore, transplantation of MSCs seems to attenuate the inflammatory response and possibly promote tissue repair and regeneration, leading to improved outcome of COVID-19 patients (Leng et al., 2020). It seems that due to the MSC-induced immunosuppression, several proinflammatory cytokines and chemokines were reduced in the serum, including IL-6 and C-reactive protein (CRP), while lymphocyte count returned to normal levels faster (Shu et al., 2020). We hypothesize that the anti-inflammatory MSC effect attenuated the recruitment of both macrophages and mononuclear cells to the inflamed lung tissue, further inducing more regulatory dendritic cells; these combined with increased IL-10 and vascular endothelial growth factor might have promoted lung repair. The effect of secreted exosomes has also been documented on antigen presentation functions, differentiation, and maturation of dendritic cells, neutrophils, and other immune cells (for details see recent reviews Abraham and Krasnodembskaya, 2020; Li et al., 2020).

These results suggest that the MSCs may help to reverse the outcome of the COVID-19 cytokine storm. Therefore, we hypothesize that the circulating fetal stem cells might have an immune-protective effect to mothers and contribute to the mild and even asymptomatic COVID-19 pregnancies.

The mechanisms of action, the life span, and the effect of circulating stem cells have been under investigation for decades. Several reports documented the presence of large numbers of fetal cells in healthy or wounded tissue (Bianchi et al., 1996; Khosrotehrani et al., 2004; Nguyen Huu et al., 2007; Zeng et al., 2010). One such study demonstrated that years after the pregnancy, presumably fetal male cells were located in pathological lung and thymus surgical specimens, and the authors speculated they were either recruited from bone marrow or had been proliferated locally (O’Donoghue et al., 2008). Moreover, the results of a gene expression analysis of fetal cells located in the murine maternal lung during pregnancy support this view (Pritchard et al., 2012). These findings back our hypothesis that the presence of placental and fetal MSCs may have an effect on prepartum and post partum maternal health.

Coronavirus disease 2019 pregnancies come forth as a paradigm to be further and more comprehensively investigated. This to understand both the mechanism and action of circulating stem cells in immune modulation and protection as well as their role in hypoxia. Non-invasive methods to monitor human pregnancies and animal models would help answer crucial aspects of both the COVID-19 disease and a possible and feasible therapeutic approaches and non-invasive treatment. When and how early fetal circulating stem cells are recruited to the lungs and what factors signal cell migration toward the maternal circulation might be answered with such studies. Moreover, this type of approaches might reveal the onset of an immunoprotective cell migration as a response to placental vascular malperfusions because of increased uterine and intervillous blood flow. Moreover, the cell migration mechanisms themselves could provide hints on blood–brain approachability for future studies and therapies.

Administration of MSCs in COVID-19 patients seems to be a promising tool in the treatment by reducing hyperinflammation (Gentile et al., 2020). MSCs from COVID-19 patients of different disease severity should be isolated and analyzed. Adipose tissue-derived stem cells (ADSCs), and their exosomes, are considered superior regarding non-invasiveness, accessibility, and abundance when compared to other sources (Jin et al., 2013). They can also be maintained and expanded in culture for long without losing their differentiation capacity, expanded thus to the large quantities needed for cell therapy purposes. ADSCs maintain their differentiation potential to differentiate, and they have low immunogenicity combined with modulatory effects, with less than 1% of them expressing human leukocyte antigen – DR isotype (HLA-DR), rendering them suitable for clinical allogeneic transplantation (Puissant et al., 2005; Strem et al., 2005; Dominici et al., 2006; McIntosh, 2011).

The route of administration and MSC preconditioning with cytokines or hypoxia should be further explored and fine-tuned. Both the COVID-19 pregnancies and the outcome of the clinical trials that investigate the possible therapeutic role of MSC transplantation will provide solid evidence of how MSCs influence the indicators of proinflammatory cytokines and further elucidate the impairment of the interferon pathway in COVID-19 patients.

Thus, studying the effect of maternal and fetal MSCS in COVID-19 pregnancies may act as a proof-of-principle approach, pave the understanding of the role of fetal circulating stem cells in the mother–fetus dyad, and elucidate the use of MSC infusion for therapy beyond COVID-19.

## Data Availability Statement

The original contributions presented in the study are included in the article/supplementary material, further inquiries can be directed to the corresponding author.

## Author Contributions

AS conceptualized the study, wrote first draft, and revised the manuscript. EH revised the manuscript. Both authors contributed to the article and approved the submitted version.

## Conflict of Interest

The authors declare that the research was conducted in the absence of any commercial or financial relationships that could be construed as a potential conflict of interest.
